# The Effect of Body Fat Distribution on Systemic Sclerosis

**DOI:** 10.3390/jcm11206014

**Published:** 2022-10-12

**Authors:** Gonzalo Villanueva-Martin, Marialbert Acosta-Herrera, Martin Kerick, Elena López-Isac, Carmen P. Simeón, José L. Callejas, Shervin Assassi, Lorenzo Beretta, International SSc Group, Australian Scleroderma Interest Group (ASIG), Yannick Allanore, Susanna M. Proudman, Mandana Nikpour, Carmen Fonseca, Christopher P. Denton, Timothy R. D. J. Radstake, Maureen D. Mayes, Xia Jiang, Javier Martin, Lara Bossini-Castillo

**Affiliations:** 1 Department of Cell Biology and Immunology, Institute of Parasitology and Biomedicine López-Neyra, CSIC, 18016 Granada, Spain; 2 Systemic Autoimmune Disease Unit, Hospital Clínico San Cecilio, Instituto de Investigación Biosanitaria Ibs. GRANADA, 18016 Granada, Spain; 3 Department of Internal Medicine, Valle de Hebrón Hospital, 08035 Barcelona, Spain; 4 Department of Internal Medicine, Hospital San Cecilio, 18016 Granada, Spain; 5 Department of Rheumatology, The University of Texas Health Science Center, Houston, TX 77030, USA; 6 Referral Center for Systemic Autoimmune Diseases, Fondazione IRCCS Ca’ Granda Ospedale Maggiore Policlinico di Milano, 20122 Milan, Italy; 7 Department of Rheumatology A, Hospital Cochin, 75005 Paris, Île-de-France, France; 8 Department of Medicine, The University of Melbourne at St. Vincent’s Hospital, Melbourne, VIC 3010, Australia; 9 Center for Rheumatology, Royal Free and University College Medical School, London 41125, UK; 10 Department of Rheumatology and Clinical Immunology, University Medical Center Utrecht, 3584 CX Utrecht, The Netherlands; 11 Department of Clinical Neuroscience, Center for Molecular Medicine, Karolinska Institutet, 17176 Stockholm, Sweden; 12 Department of Epidemiology, Harvard T.H. Chan School of Public Health, Boston, MA 02115, USA; 13 West China School of Public Health, West China Fourth Hospital, Sichuan University, Chengdu 610041, China; 14 Departamento de Genética e Instituto de Biotecnología, Centro de Investigación Biomédica (CIBM), Universidad de Granada, 18100 Granada, Spain; 15 Advanced Therapies and Biomedical Technologies (TEC-14), Instituto de Investigación Biosanitaria Ibs. GRANADA, 18016 Granada, Spain

**Keywords:** systemic sclerosis, mendelian randomization, obesity

## Abstract

Obesity contributes to a chronic proinflammatory state, which is a known risk factor to develop immune-mediated diseases. However, its role in systemic sclerosis (SSc) remains to be elucidated. Therefore, we conducted a two-sample mendelian randomization (2SMR) study to analyze the effect of three body fat distribution parameters in SSc. As instrumental variables, we used the allele effects described for single nucleotide polymorphisms (SNPs) in different genome-wide association studies (GWAS) for SSc, body mass index (BMI), waist-to-hip ratio (WHR) and WHR adjusted for BMI (WHRadjBMI). We performed local (pHESS) and genome-wide (LDSC) genetic correlation analyses between each of the traits and SSc and we applied several Mendelian randomization (MR) methods (i.e., random effects inverse-variance weight, MR-Egger regression, MR pleiotropy residual sum and outlier method and a multivariable model). Our results show no genetic correlation or causal relationship between any of these traits and SSc. Nevertheless, we observed a negative causal association between WHRadjBMI and SSc, which might be due to the effect of gastrointestinal complications suffered by the majority of SSc patients. In conclusion, reverse causality might be an especially difficult confounding factor to define the effect of obesity in the onset of SSc.

## 1. Introduction

Systemic sclerosis (SSc) is an immune-mediated disease (IMD), characterized by abnormal immunological activation, vascular damage and fibrosis of the skin [1]. SSc represents a major challenge for clinicians, as it has a deep impact on the life quality and life expectancy of the affected patients [1]. Recent efforts in the study of the genetic factors that contribute to the onset and progression of SSc, such as several large-scale genetic association studies and genome-wide association studies (GWAS) [2], have contributed to identifying genetic susceptibility markers both in the human leukocyte antigen (HLA) locus and outside this highly polymorphic region [3]. The largest GWAS to date comprised more than 9000 patients with SSc and allowed the identification of 19 non-HLA loci associated with the disease [2]. Moreover, recent studies have identified specific *HLA-DQA1* alleles exclusively associated with different clinical subtypes of SSc [3]. Therefore, the number of relevant loci that have been firmly associated with this condition has remarkably increased over the last decade. Although the use of genetic risk factors to predict the risk of developing SSc was explored in a recent genomic risk score (GRS) [4], the involvement of these genetic risk factors in the disease pathogenesis and the affected biological pathways have not been fully established yet [5].

Despite the advances in the identification of the genetic factors contributing to the heritability of SSc, the complex nature of this disorder is an intrinsic obstacle to studying the pathological mechanisms that lead to the disruption of the immune homeostasis and to the onset of fibrotic processes in affected individuals. Well-established environmental triggers for SSc are silica and solvents, extreme or long-term exposure to which is related to the disease’s development [6,7]. Moreover, demographic and clinical characteristics, such as sex, age, ethnical origin, hormone levels, etc., have been pointed out as risk factors for SSc [6,8]. However, the roles of lifestyle and environmental triggers in the manifestation and prognosis of SSc are still elusive.

Mendelian randomization (MR) uses SNPs as instrumental variants (IVs) in order to determine if they are acting on a disease or outcome through a risk factor or exposure [9,10]. The principle of the method is that alleles are randomly distributed during gametogenesis and their presence pre-exists the disease. These genetic facts mimic the random distribution of clinical trials and take away the causality of the disease on the variable, reducing confounding factors [11]. For a genetic variant to be considered an IV, it is assumed that it is associated with exposure. However, an IV cannot be associated with any confounding factor related to the risk factor or the outcome, either directly or indirectly. Additionally, the effects of the IV on the outcome should only be mediated by the exposure [9]. Therefore, only when genetic polymorphisms are relevant, independent and have a restricted effect on the outcome, can they be considered IVs. In a classical MR study, the allele effects on outcome and exposure are obtained from the same individuals [9,10]. However, detailed information for multiple traits is difficult to obtain in a large population. Two-sample MR (2SMR) methods allow us to combine the estimations of the IV allele effects relying only on GWAS summary statistics for the outcome and for the exposure from independent studies. The implementation of these methods has improved the statistical power to detect causal associations between risk factors and disease, which has shown promising results in several conditions [12].

Obesity-related diseases are becoming a public health issue in Western countries [13], since obesity rates are increasing due to unhealthy lifestyles. Obesity is defined by an excess of fat in the body and body fat distribution can be measured by a variety of methods, for instance body mass index (BMI) and waist to hip ratio (WHR). BMI is the most common body fat proxy and it is the gold standard for obesity. BMI is measured as the body weight normalized by height squared (kg/m^2^) [14], and it is known that BMI > 25 kg/m^2^ is associated with an increased risk of suffering from chronic diseases such as cardiovascular disease, type II diabetes or specific cancers [15]. Nevertheless, BMI has certain limitations, and anthropometric measures of abdominal obesity, such as WHR, seem to be better indicators of excessive fat mass [16]. Since WHR measures both visceral and gluteal fat, it stands out among other anthropometric traits [17]. If WHR is adjusted for BMI (WHRadjBMI), it is possible to obtain an anthropometric measure which is independent from the overall adiposity, and to combine the most standardized measure of obesity and the anthropometric measure that best captures the distribution of body fat [17,18]. Taking advantage of the publicly available GWAS results, MR approaches have been successful in identifying risk factors for IMDs, such as obesity-related traits [19,20]. Excess of fat has been associated with a low but persistent proinflammatory state that is believed to promote IMDs [13,21]. However, in the case of SSc, the relationship between body fat distribution and SSc remains to be explored.

Consequently, in order to analyze the effect of nutritional status on SSc risk, we applied the novel 2SMR methods on the largest GWAS of SSc patients [2] with European ancestry and the biggest GWAS meta-analysis for anthropometric fat distribution measures to date [22].

## 2. Materials and Methods

### 2.1. Instrumental Variables

**Figure 1:**
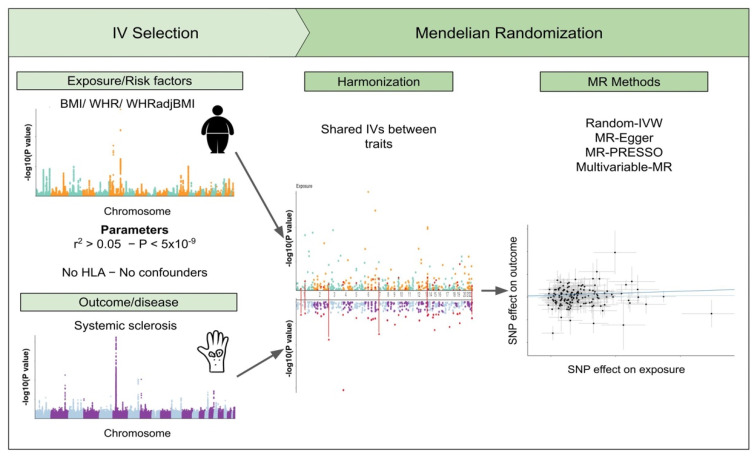
Schematic representation of the study design. The study is divided into several phases, i.e., selection of the instrumental variables for the outcome and the exposures, data harmonization and generation of different Mendelian randomization models.

The study design of the 2SMR study of SSc and three obesity-related traits is summarized in Figure 1. The outcome instrumental variables (IV-outcome), i.e., the selected genetic variants and their effect sizes in SSc, were obtained from the largest SSc GWAS meta-analysis, which included 9846 SSc patients and 18,333 healthy controls from 14 different cohorts with European ancestry [2]. Patient demographic data are shown in Table S1. Additionally, SNP effect sizes after stratification by sex, serological and clinical subtype, as reported elsewhere [3], were also analyzed. Finally, we performed sex-specific analyses, including only either the female or the male individuals from the different cohorts and following the previously described analysis framework [2].

In the case of the exposures, we obtained the IVs (IV-exposure) from a recent GWAS meta-analysis between the cohorts included in the Genetic Investigation of Anthropometric Traits consortium (GIANT) project and those recruited for the UK Biobank (UKBB) repository for different anthropometric measures [23]. We only used the summary statistics comprising individuals with European ancestry, which included 806,810 individuals and 27,381,302 SNPs for BMI, a classical obesity parameter, and for two parameters that assess body fat distribution: WHR, comprising 697,734 individuals and 27,376,273 SNPs, and WHRadjBMI, covering 694,649 individuals and 27,375,636 SNPs [23]. None of the participants recruited in the SSc studies overlapped with the exposure GWASs to the best of our knowledge.

### 2.2. Genomic Association Analysis

Genetic correlation. To determine causality between obesity risk factors and SSc, we calculated the total genomic correlation between them. First, we performed an approximation implemented in the linkage disequilibrium regression score (LDSC) software [24]. Then, to study the contribution of specific regions (pairwise local genetic correlation), we used the methods supported by ρ-HESS software (version 0.5.3) [25]. Briefly, ρ-HESS software splits the genome into 1703 small regions through the chromosomes and uses LD matrices to create eigenvectors and to project the GWAS effect sizes. Then, the local SNP heritability per trait is calculated and, finally, genetic covariance between traits is estimated. We adjusted our significance thresholds for multiple testing, i.e., 1.1 × 10^−3^ (0.05/45) for LDSC and 2.9 × 10^−5^ (0.05/1703) for ρ-HESS.

Mendelian randomization analysis. In order to assess if there was a causal relationship between body fat distribution and SSc or any of the stratified sets of patients, we performed a 2SMR study implementing the R package “TwoSampleMR” [10]. Considering the complex linkage disequilibrium (LD) patterns and the strong genetic associations described in the HLA locus SSc [2,3,26], the extended HLA region (chromosome 6: 20,000,000–40,000,000 bp) was excluded from the MR analyses in order to prevent biases. 

The selected IVs were based on the original independent signal analysis reported by Pulit et al. [23]. Briefly, the independent signals from results from the inverse variance meta-analysis (*p* < 5 × 10^−9^) were identified by LD-based clumping (r^2^ > 0.05 and ±5 Mb). Secondary signals were also defined by conditional analyses (*p* < 5 × 10^−9^) and locus LD clumping. We extracted the association estimates for these SNPs or the best available proxy (according to the LD patterns observed in the UKBB cohort) that was present in the SSc dataset. The number of shared SNPs between SSc and the exposures reached 533, 247 and 262 for BMI, WHR and WHRadjBMI, respectively (Table S2). 

Three gold-standard 2SMR methods were selected. A random effects inverse-variance weight (IVW) approach was taken, which pools the effects of each IV and balances the global pleiotropy to zero by assuming the validity or invalidity of all the SNPs [10]. An MR-Egger regression method [27] was applied, which is able to estimate causality even when all IVs are weak or invalid and to calculate horizontal pleiotropy. Although these methods are very robust for MR analysis, both of them have limitations in dealing with outlier IVs. For that reason, we also applied the MR pleiotropy residual sum and outlier (MR-PRESSO) method [28]. The MR-PRESSO algorithm detects outlier IVs that exert horizontal pleiotropy in a multi-instrument Mendelian randomization analysis. Moreover, MR-PRESSO provides outlier-free causality estimates.

Additionally, to estimate the effect of the IVs controlling for their effect on other exposures, we performed a multivariable Mendelian randomization analysis (MVMR), implemented in the TwoSampleMR package [29]. This analysis included a set of unique LD-clumped IV exposures for both BMI and WHR, which were regressed against SSc together, weighting for the inverse variance of SSc for these IVs. 

The Benjamini–Hochberg false discovery rate (FDR) correction was applied, and we considered *p* < 0.05 as significant [30].

### 2.3. Sensitivity Analysis

The statistical power of our analyses was calculated using the algorithm described by Brion et al. for MR studies [31]. Aiming to control for the effect of potential confounding factors, we removed any of the SNPs with reported associations with known obesity-related confounding factors (Table S3) from the MR analysis, as reported by the GWAS catalog [32], SNPnexus [33] and ClinVar [34]. We studied the contribution of each SNP to the observed effects by carrying out a leave-one-out sensitivity analysis, implemented in the “TwoSampleMR” package [10]. By these means, we observed that the exclusion of one SNP at a time did not affect the observed results.

## 3. Results

Leveraging Mendelian randomization as a novel methodological strategy, we studied for the first time the causal contribution of body fat distribution to the risk of suffering from SSc (Figure 1). Here we used the GWAS summary statistics of the largest SSc meta-analysis [2] as an outcome and three obesity-related traits GWAS comprising thousands of European ancestry individuals as exposures.

### 3.1. Genomic Correlation. Only the HLA Locus Harbours Local Genetic Correlation between SSc and Body Fat Distribution

**Figure 2:**
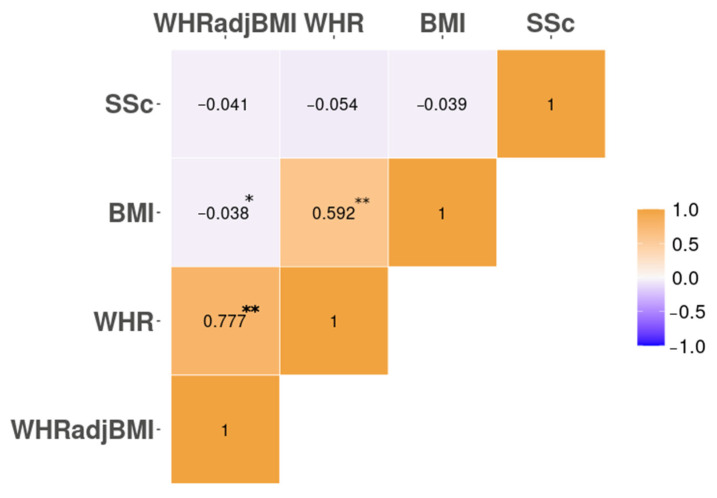
Pairwise global genetic correlation observed between the three obesity-related exposures and SSc. * = *p* > 0.05 (suggestive for statistical significance); ** = *p* > 0.00625 (Bonferroni-corrected).

At a genomic scale, we observed a strong genome-wide correlation between BMI and WHR (rg = 0.59, [95% CI −0.016–0.051]) and between WHR and WHRadjBMI (rg = 0.78, [95% CI −0.01–0.03]), but not between WHRadjBMI and BMI (rg = −4.02 × 10^−2^, [95% CI −0.016–0.049]), as previously described [19] (Figure 2). However, our results show no evidence of correlation between SSc and the three tested obesity-related traits (BMI rg = −0.039 [95% CI −0.033–0.102]; WHR rg = −0.054, [95% CI −0.035–0.106]; WHRadjBMI rg = −0.041, [95% CI −0.04–0.122], all observed *p* > 0.05) (Figure 2).

**Figure A1:**
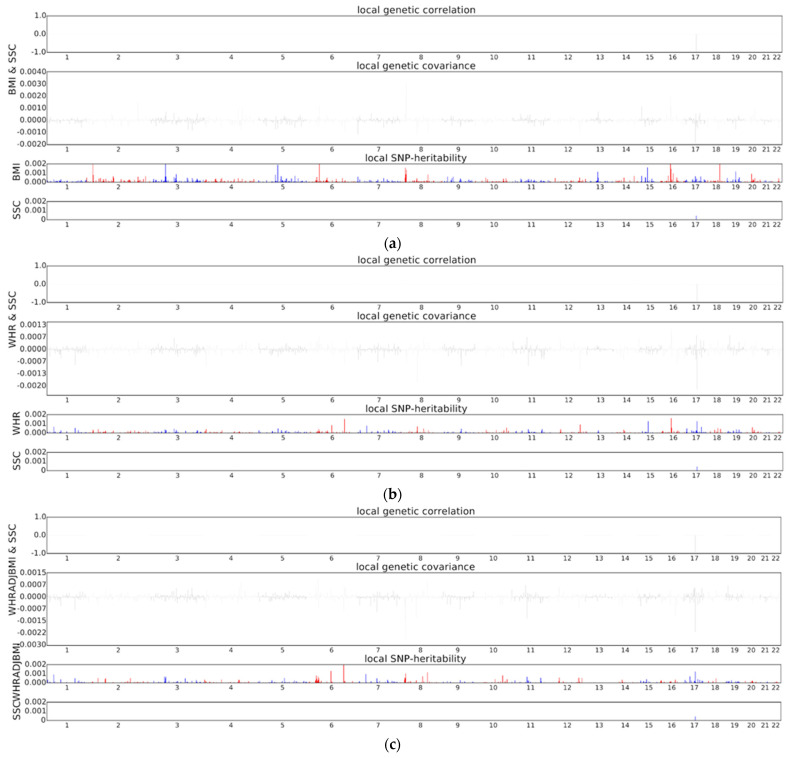
Local genetic correlation, local genetic variance and local SNP heritability between SSc and (**a**) BMI, (**b**) WHR and (**c**) WHRadjBMI.

Even when there is no correlation between traits at a genome-wide level, it is possible that the traits show local correlation at specific loci. To address this potential correlation, we performed a local genetic correlation analysis between BMI, WHR, WHRadjBMI and SSc (Figure A1). The local correlation observed in these regions reached local-rg = 8.5 × 10^−4^ and local-rg = 2.6 × 10^−4^ (Figure A1).

### 3.2. The Analysis of the Causal Relationship between Obesity-Related Traits and Systemic Sclerosis Is Limited by Confounding Factors

Despite the limited genetic correlation found, we explored the possible causal relationship between body fat distribution and SSc. Considering the complex LD patterns in the HLA regions and the local genetic correlation found only in this locus, it was excluded from the following MR analyses. The available SSc dataset was powerful enough to detect associations of 25% increased risk of SSc with BMI (99%), WHR (83%) and WHRadjBMI (92%) (Table S4), considering an explained phenotypic variance of 2.5–5% and the complete set comprising 28,179 individuals (34.9% cases). We were confident about the statistical power estimated for the largest subsets of patients, for instance, females (BMI power = 79%, WHR power = 82% and WHRadjBMI = 87%), lcSSc (BMI power = 94%, WHR power = 70% and WHRadjBMI = 81%) and ACA+ (BMI power = 83%, WHR power = 53% and WHRadjBMI = 65%). However, the analyses for the less frequent patient groups, i.e., males (BMI power = 30%, WHR power = 8% and WHRadjBMI = 10%) and ATA+ (BMI power = 14%, WHR power = 10% and WHRadjBMI = 12%) were clearly insufficient to identify true causal relationships (Table S4).

As reported in Table 1 and Table S5, classical MR methods showed no significant evidence of causality for BMI or WHR on SSc, whether including only the index SNPs or considering both the index SNPs and the secondary signals. The results for BMI under the random effects IVW model show a suggestive positive association with BMI, but this association did not reach statistical significance (OR under random effects IVW = 1.15 [95% CI 0.67–1.98]). A trend of negative association considering index and secondary signals was only observed in the case of the random effects IVW model for WHR (Table 1). All the remaining models showed *p* > 0.05 and the ORs were in the range of 0.93–1.15 for BMI and 0.27–0.82 for WHR. In the case of WHRadjBMI (WHR after regressing out the effect of BMI), a negative association with SSc reached statistical significance in the three tested models (OR under random effects IVW = 0.73 [95% CI 0.56–0.94], MR-Egger = 0.43 [95% CI 0.20–0.90], MR-PRESSO = 0.77 [95% CI 0.60–0.99]). These associations with WHRadjBMI remained negative in the analyses that included only index signals, but only the MR-Egger model was significant after multiple testing correction (OR under MR-Egger = 0.69 [95% CI 0.51–0.93], see Table S5).

**Figure A2:**
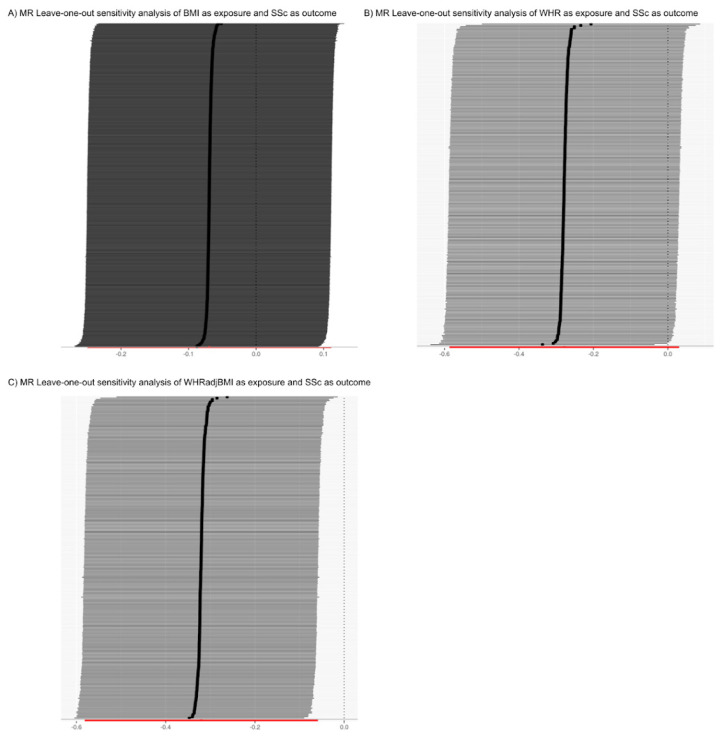
MR leave-one-out sensitivity analyses for: (**A**) BMI, (**B**) WHR and (**C**) WHRadjBMI.

We carried out a sensitivity analysis, which implied the removal of SNPs associated with known obesity-related confounders (Table S3), to address the effect of these confounders on the lack of significance for the BMI models and the negative relationships with WHR and WHRadjBMI. As shown in Table 2 and Table S6, the confounder-free models did not change the observed negative relationship and none of them reached a significant result after FDR correction. Although we observed effect size heterogeneity for the different genetic variants (Table S7), the analyses of the intercept parameter in the MR-Egger models did not reveal any signs of horizontal pleiotropy, and the effects were not affected by the removal of the outlier SNPs identified by the MR-PRESSO algorithm (Table 1 and Table 2, Tables S5 and S6). Furthermore, leave-one-out analyses did not highlight that these effects were influenced only by one variant (Figure A2).

We decided to implement an MVMR model, considering the significant associations observed for WHRadjBMI and the limitations of the univariate models, to test for the combined influence of several exposures and to control for the effect of confounding factors. This analysis allowed us to directly test the association of BMI and WHR with SSc, controlling for the effects of both parameters at the same time. As expected, the results of these analyses show an effect for WHR (MVMR OR 0.80 [95% CI 0.57–1.13]) that is similar to the previously identified effect for WHRadjBMI (Table 3). Nevertheless, no significant association of BMI with SSc was revealed (MVMR OR 1.03 [95% CI 0.79–1.33]) (Table 3). These findings might point towards a negative or inexistent effect of WHR in SSc and, if any, a very modest risk effect for BMI.

Considering the well-known clinical and genetic differences between the SSc subsets of patients [35], we explored subset-specific effects for the selected exposures. Several associations remained significant in the stratified analyses, especially in the largest and more powerful subsets, such as lcSSc (Table S8). However, the direction and magnitude of the exposure effects were consistent in all the subsets (Table S8), which suggested a uniform effect, if any, in all the patients. There were no significant differences between the models with and without the secondary signals (Table S8). Moreover, taking into account the higher frequency of SSc in females (9 female: 1 male ratio) [8], we performed sex-specific analyses too. In these analyses, we relied on female-only and male-only GWAS summary statistics for both SSc and the obesity-related risk factors. Once more, although the risk effect of BMI, WHR and WHRadjBMI seemed more evident in men, these effects did not reach statistical significance (Table S8).

## 4. Discussion

This report addressed the risk effect of body fat distribution in SSc for the first time. We exhaustively exploited public GWAS summary statistics for both SSc and for anthropometric traits and the development of novel MR methods. We did not observe global genomic correlation between the outcome and any of the exposures. Moreover, local genetic correlation was only found in the HLA locus, a highly complex region. Different MR methods were then applied to identify possible causal relationships between the obesity traits and SSc. However, no significant causal risk effect of the exposures was found in this case.

Although our results do not support the causal relation between exposures and outcome, it should be noted that the statistical power of the SSc dataset is modest compared to similar studies performed to date in other IMDs, such as RA or IBD [36] (Table S2). SSc is a rare IMD and, despite recent advances [1,2,26], the recruitment of large patient cohorts remains challenging. Therefore, future efforts to enlarge the size or to complement the available SSc GWAS information might help to identify causal risk factors.

We found that the effect of confounders might be more severe in the case of SSc than in other IMDs. Gastrointestinal involvement (GI), which affects more than 70% of SSc patients [36], hinders food ingestion, and patients are mostly thin [37]. In fact, weight loss has been used as one of the SSc diagnostic markers [35]. This direct effect of the onset symptoms in the exposures is known as reverse causality, and it is a remarkably difficult confounding factor to control for [38]. Reverse causality might be the cause behind both the lack of significant risk effects of BMI in SSc and the reported negative relationship between WHR and SSc, which becomes more evident when the effect of BMI is subtracted in the analysis of WHRadjBMI (Table 1 and Table 2, Table S5 and Table S6).

Bad diet habits and obesity are associated with an increased risk to suffer from IMDs such as RA and IBD [19,20,39]. Higher BMI has been associated with increased risk to Crohn’s disease (CD) and Rheumatoid Arthritis (RA), but negative associations with BMI have been reported for ulcerative colitis (UC), and a recent study found reverse causality between WHR and RA [19,20,39]. IMDs are often present as comorbidities and share altered molecular pathways, environmental triggers and genetic risk factors [40]. Furthermore, the role of adipocytes in the activation of the immune system is prominent, especially due to the release of adipokines [41]. Adipokines are molecules known to be involved in the “obesity–autoimmunity” relationship [13,42], such as lectins or cytokines, especially adiponectin, but also interleukins and tumor necrosis factor alpha (TNFɑ) [13]. Interestingly, patients with SSc and a high BMI have been shown to have higher lectin levels than healthy controls [43], and it has been established that subcutaneous adipocytes can act as progenitor cells for fibroblasts [44,45]. These fibroblasts may eventually transdifferentiate into myofibroblasts [46], activated profibrotic fibroblasts that are characteristic of the fibrotic lesions observed in SSc patients, and recent evidence has shown that the activation of adipocyte-derived mesenchymal cells from SSc skin biopsies to myofibroblasts is possible using soluble molecules present in the skin microenvironment in SSc [47].

In order to rule out the role of obesity as a risk factor for SSc, body fat distribution measures from the patients before the onset of GI or BMI-matched case-control sets would be very valuable resources.

The negative association that is observed for WHR might be due to additional confounding factors that are inherent to SSc and that affect body fat distribution, for example, sex or lipid profiles [15]. Remarkably, WHR is different in women than in men and there is a clear sex bias in SSc [35]. Therefore, we hypothesized that there could be a sex-specific association and performed stratified analyses with the female and male cohorts separately. Our results show significant causal associations with SSc only in females, but considering the statistical power differences and the similarity between the effect sizes, the lack of significance for the male group may be likely due to the reduced sample size (Table S4). The key role of sample size as a limitation of our study to identify weak risk effects was also clear in other stratified analyses, as we found consistent ORs for all the tested clinical subtypes of SSc patients, but the models reached statistical significance only in the largest subsets (Table S8).

In conclusion, this study found no significant evidence that supported the role of body fat distribution as a causal risk factor for SSc using 2SMR methods. Nevertheless, the current GWAS have a limited statistical power to identify modest contributions to SSc risk and the intrinsic nature of the SSc clinical complications might be acting as potential constraints in this study. Consequently, further analyses are needed to rule out the role of obesity in the onset of Ssc.

## Figures and Tables

**Table 1:** Association between genetically predicted obesity-related traits and risk of SSc. Analysis including index and secondary signals for the obesity-related traits and excluding the HLA region. BMI: body mass index, WHR: waist to hip ratio, WHRadjBMI: WHR adjusted for BMI, MR: Mendelian randomization, nSNPs: number of single nucleotide polymorphisms, OR: Odds Ratio, CI: confidence interval, *p*: *p* value, *p* adj: *p* value after FDR correction for multiple testing, IVW: inverse-variance weight, PRESSO: pleiotropy residual sum and outlier, NA: not applicable.

	**Index and secondary SNPs (*p* < 5 × 10^−9^)**					
	**MR Approach**	**nSNPs**	**OR (95% CI)**	***p***	***p*** **adj**	***p*** **for Heterogeneity or Pleiotropy**
						
**BMI**	MR-Egger	533	1.0575(0.6403–1.7466)	0.827	0.8273	0.6005
	Random-effects IVW		0.9326(0.7787–1.117)	0.449	0.4485	<0.001
	MR-PRESSO (1) *		0.943(0.7892–1.1269)	0.5189	NA	NA
						
**WHR**	MR-Egger	247	0.2698(0.0914–0.7965)	0.0185	0.0384	0.0519
	Random-effects IVW		0.7564(0.5567–1.0278)	0.0743	0.11145	<0.001
	MR-PRESSO (3) *		0.7809(0.5907–1.0324)	0.0838	NA	NA
						
**WHRadjBMI**	MR-Egger	262	0.4251(0.2014–0.8971)	0.0256	0.0384	0.1344
	Random-effects IVW		0.7269(0.5603–0.9431)	0.0163	0.0489	<0.001
	MR-PRESSO (1) *		0.77(0.6015–0.9857)	0.039	NA	NA

* Number of outlier SNPs detected by MR-PRESSO.

**Table 2:** Association between genetically predicted obesity-related traits and risk of SSc. Analysis including index and secondary signals for the obesity-related traits and excluding the HLA region and known obesity-related confounder SNPs. BMI: body mass index, WHR: waist to hip ratio, WHRadjBMI: WHR adjusted for BMI, MR: Mendelian randomization, nSNPs: number of single nucleotide polymorphisms, OR: odds ratio, CI: confidence interval, *p*: *p* value, *p* adj: *p* value after FDR correction for multiple testing, IVW: inverse-variance weight, PRESSO: pleiotropy residual sum and outlier, NA: not applicable.

	**Index and Secondary SNPs (*p* < 5 × 10^−9^)**					
	**MR Approach**	**nSNPs**	**OR (95% CI)**	***p***	***p*** **adj**	***p*** **for Heterogeneity or Pleiotropy**
						
**BMI**	MR-Egger	483	1.422(0.721–2.803)	0.3103	0.3103	0.1769
	Random-effects IVW		0.909(0.741–1.115)	0.3598	0.3598	0.0011
	MR-PRESSO (1) *		0.922(0.754–1.128)	0.4288	NA	NA
						
**WHR**	MR-Egger	221	0.301(0.086–1.060)	0.0629	0.09435	0.1391
	Random-effects IVW		0.752(0.535–1.057)	0.1007	0.15105	< 0.001
	MR-PRESSO (2) *		0.764(0.559–1.044)	0.0927	NA	NA
						
**WHRadjBMI**	MR-Egger	237	0.335(0.137–0.819)	0.0172	0.0516	0.0772
	Random-effects IVW		0.716(0.534–0.961)	0.0261	0.0783	< 0.001
	MR-PRESSO (1) *		0.769(0.582–1.015)	0.0651	NA	NA

* Number of outlier SNPs detected by MR-PRESSO.

**Table 3:** Multivariable MR (MVMR) model including BMI. WHR and risk of SSc. Analysis including index and secondary signals for the obesity-related traits and excluding the HLA region, with and without known obesity-related confounding SNPs. BMI: body mass index, WHR: waist to hip ratio, MR: Mendelian randomization, nSNPs: number of single nucleotide polymorphisms, OR: odds ratio, CI: confidence interval, *p*: *p* value.

**Before Confounder SNP Removal.**					**After Confounder SNP Removal**				
Index and secondary SNPs (*p* < 5 × 10^−9^)					Index and secondary SNPs (*p* < 5 × 10^−9^)				
**Outcome**	**Exposure**	**nSNP**	**OR (95% CI)**	***p***	**Outcome**	**Exposure**	**nSNP**	**OR (95% CI)**	***p***
SSc	BMI	666	1.026(0.79–1.331)	0.849	SSc	BMI	610	1.027(0.760–1.387)	0.863
	WHR	666	0.804(0.573–1.128)	0.207		WHR	610	0.812(0.552–1.195)	0.291
Index SNPs (*p* < 5 × 10^−9^)					Index SNPs (*p* < 5 × 10^−9^)				
**Outcome**	**Exposure**	**nSNP**	**OR (95% CI)**	***p***	**Outcome**	**Exposure**	**nSNP**	**OR (95% CI)**	***p***
SSc	BMI	581	0.99(0.749–1.309)	0.946	SSc	BMI	524	1.013(0.726–1.412)	0.941
	WHR	581	0.876(0.607–1.263)	0.477		WHR	524	0.881(0.574–1.352)	0.561

## Supplementary Materials

The following supporting information can be downloaded at: https://www.mdpi.com/article/10.3390/jcm11206014/s1. Table S1. Demographic data from SSc meta GWAS. Table S2. GWAS selection summary. Table S3. List of discarded SNPs due to association with known obesity-related confounders. Table S4. Statistical power calculation for each stratification and outcome-exposure combination. Table S5. Association between genetically-predicted obesity-related traits and risk of SSc. Analysis including index and secondary signals for the obesity-related traits and excluding the HLA region. Table S6. Association between genetically-predicted obesity-related traits and risk of SSc. Analysis including index signals for the obesity-related traits and excluding the HLA region and known obesity-related confounder SNPs. Table S7. Effect sizes in the exposures and in the outcome of the BMI, WHR and WHRadjBMI index and secondary SNPs. Table S8. Association between genetically-predicted obesity-related traits and risk of SSc. Analysis including index and secondary signals for the obesity-related traits and excluding the HLA region and known obesity-related confounder SNPs. SSc stratifications comprise sex (females and males), clinical subtypes (lcSSc and dcSSc) and serological subsets (ACA+ and ATA+). File S1: Collaborators.
