# Supplementary material for: The Effect of Body Fat Distribution on Systemic Sclerosis
Source: J Clin Med. 2022 Oct 12;11(20):6014. doi: 10.3390/jcm11206014 (PMC9604859; doi:10.3390/jcm11206014)
Supplement: Supplementary file 1 [file jcm-11-06014-s001.zip › jcm-1885654-Collaborators.pdf]

Collaborators:

International SSc Group:

P. Carreira, Department of Rheumatology, 12 de Octubre University Hospital, Madrid, Spain; I. Castellvi, Department of Rheumatology, Santa Creu i Sant Pau University Hospital, Barcelona, Spain; R. Ríos, Department of Internal Medicine, San Cecilio Clinic University Hospital, Granada, Spain; N. Ortego-Centeno, Department of Medicine, University of Granada, Granada, Spain; R. García Portales, Department of Rheumatology, Virgen de la Victoria Hospital, Málaga, Spain; A. Fernández-Nebro, Department of Rheumatology, Carlos Haya Hospital, Málaga, Spain; F. J. García-Hernández, Department of Internal Medicine, Virgen del Rocío Hospital, Sevilla, Spain; M. A. Aguirre, Department of Rheumatology, Reina Sofía/IMIBIC Hospital, Córdoba, Spain; B. Fernández-Gutiérrez, Department of Rheumatology, San Carlos Clinic Hospital, Madrid, Spain; L. Rodríguez-Rodríguez, Department of Rheumatology, San Carlos Clinic Hospital, Madrid, Spain; P. García de la Peña, Department of Rheumatology, Madrid Norte Sanchinarro Hospital, Madrid, Spain; E. Vicente, Department of Rheumatology, La Princesa Hospital, Madrid, Spain; J. L. Andreu, Department of Rheumatology, Puerta de Hierro Hospital-Majadahonda, Madrid, Spain; M. Fernández de Castro, Department of Rheumatology, Puerta de Hierro Hospital-Majadahonda, Madrid, Spain; F. J. López-Longo, Department of Rheumatology, Gregorio Marañón University Hospital, Madrid, Spain; V. Fonollosa, Department of Internal Medicine, Valle de Hebrón Hospital, Barcelona, Spain; A. Guillén, Department of Internal Medicine, Valle de Hebrón Hospital, Barcelona, Spain; G. Espinosa, Department of Internal Medicine, Clinic Hospital, Barcelona, Spain; C. Tolosa, Department of Internal Medicine, Parc Tauli Hospital, Sabadell, Spain; A. Pros, Department of Rheumatology, Hospital Del Mar, Barcelona, Spain; E. Beltrán, Department of Rheumatology, Hospital Del Mar, Barcelona, Spain; M. Rodríguez Carballeira, Department of Internal Medicine, Hospital Universitari Mútua Terrasa, Barcelona, Spain; F. J. Narváez, Department of Rheumatology, Bellvitge University Hospital, Barcelona, Spain; M. Rubio Rivas, Department of Internal Medicine, Bellvitge University Hospital, Barcelona, Spain; V. Ortiz-Santamaría, Department of Rheumatology, Granollers Hospital, Granollers, Spain; A. B. Madroñero, Department of Internal Medicine, Hospital General San Jorge, Huesca, Spain; M. A. González-Gay, Epidemiology, Genetics and Atherosclerosis Research Group on Systemic Inflammatory Diseases, IDIVAL, University of Cantabria, Santander, Spain; B. Díaz, Department of Internal Medicine, Hospital Central de Asturias, Oviedo, Spain; L. Trapiella, Department of Internal Medicine, Hospital Central de Asturias, Oviedo, Spain; M. V. Egurbide, Department of Internal Medicine, Hospital Universitario Cruces, Barakaldo, Spain; P. Fanlo-Mateo, Department of Internal Medicine, Hospital Virgen del Camino, Pamplona, Spain; L. Saez-Comet, Department of Internal Medicine, Hospital Universitario Miguel Servet, Zaragoza, Spain; F. Díaz, Department of Rheumatology, Hospital Universitario de Canarias, Tenerife, Spain; J. A. Roman-Ivorra, Department of Rheumatology, Hospital Universitari i Politecnic La Fe, Valencia, Spain; J. J. Alegre Sancho, Department of Rheumatology, Hospital Universitari Doctor Peset, Valencia, Spain; M. Freire, Department of Internal Medicine, Thrombosis and Vasculitis Unit, Complexo Hospitalario Universitario de Vigo, Vigo, Spain; F. J. Blanco Garcia, Department of Rheumatology, INIBIC-Hospital Universitario A Coruña, La Coruña, Spain; N. Oreiro, Department of Rheumatology, INIBIC-Hospital Universitario A Coruña, La Coruña, Spain; T. Witte, Department of Clinical Immunology, Hannover Medical School, Hannover, Germany; A. Kreuter, Department of Dermatology, Josefs-Hospital, Ruhr University Bochum, Bochum, Germany; G. Riemekasten, Clinic of Rheumatology, University of Lübeck, Lübeck, Germany; P. Airo, Service of Rheumatology and Clinic Immunology Spedali Civili, Brescia, Italy; C. Magro, Department of Rheumatology, Leiden University Medical Center, Leiden, The Netherlands; A. E. Voskuyl, Department of Rheumatology, VU University Medical Center, Amsterdam, The Netherlands; M. C. Vonk, Department of Rheumatology, Radboud University Nijmegen Medical Center, Nijmegen, Netherlands; R. Hesselstrand, Department of Rheumatology, Lund University, Lund, Sweden; A. Nordin, Division of Rheumatology, Department of Medicine, Karolinska University Hospital, Karolinska Institute, Stockholm, Sweden; C. Lunardi, Department of Medicine, Università degli Studi di Verona, Verona, Italy; G. Moroncini, Department of Clinical and Molecular Science, Università Politecnica delle Marche and Ospedali Riuniti, Ancona, Italy; A. Gabrielli, Istituto di Clinica Medica Generale,

Ematologia ed Immunologia Clinica, Università Politecnica delle Marche, Ancona, Italy; A. Hoffmann-Vold, Department of Rheumatology, Oslo University Hospital, Oslo, Norway; J. H. W. Distler, Department of Internal Medicine 3, Institute for Clinical Immunology, University of Erlangen-Nuremberg, Erlangen, Germany; L. Padyukov, Division of Rheumatology, Department of Medicine, Karolinska University Hospital, Karolinska Institute, Stockholm, Sweden; B. P. C. Koeleman, University Medical Center Utrecht, Utrecht, The Netherlands.

Australian Scleroderma Interest Group (ASIG)
W. Stevens, St. Vincent's Hospital, Melbourne, Victoria, Australia; M. Nikpour, The University of Melbourne at St. Vincent's Hospital, Melbourne, Victoria, Australia; J. Zochling, Menzies Research Institute Tasmania, University of Tasmania, Hobart, TAS, Australia; J. Sahhar, Department Rheumatology, Monash Medical Centre, Melbourne, VIC, Australia; J. Roddy, Rheumatology, Royal Perth Hospital, Perth, WA, Australia; P. Nash, Research Unit, Sunshine Coast Rheumatology, Maroochydore, QLD, Australia; K. Tymms, Canberra Rheumatology, Canberra, ACT, Australia; M. Rischmueller, Department Rheumatology, The Queen Elizabeth Hospital,Woodville, SA, Australia; S. Lester, Department Rheumatology, The Queen Elizabeth Hospital, Woodville, SA, Australia.
